# Recombinant measles virus vaccine rMV-Hu191 exerts an oncolytic effect on esophageal squamous cell carcinoma via caspase-3/GSDME-mediated pyroptosis

**DOI:** 10.1038/s41420-023-01466-2

**Published:** 2023-05-19

**Authors:** Ailing Wu, Zhongyue Li, Yilong Wang, Yi Chen, Jinkai Peng, Mengying Zhu, Yueyue Li, Hai Song, Dongming Zhou, Chudi Zhang, Yao Lv, Zhengyan Zhao

**Affiliations:** 1 Children’s Hospital, Zhejiang University School of Medicine, National Clinical Research Center for Child Health, Hangzhou, China; 2 Zhejiang University School of Medicine, Hangzhou, China; 3 Zhejiang Biosan Biotechnology Co., Ltd., Hangzhou, China; 4 The MOE Key Laboratory of Biosystems Homeostasis & Protection, Zhejiang Provincial Key Laboratory for Cancer Molecular Cell Biology and Innovation Center for Cell Signaling Network, Life Sciences Institute, Zhejiang University, Hangzhou, China; 5 Department of Thoracic Surgery, Second Affiliated Hospital, School of Medicine, Zhejiang University, Hangzhou, China

**Keywords:** Cell signalling, Gastrointestinal cancer

## Abstract

Oncolytic viruses have recently been proven to be an effective and promising cancer therapeutic strategy, but there is rare data about oncolytic therapy in esophageal squamous cell carcinoma (ESCC), especially oncolytic measles virotherapy. Therefore, this study aimed to explore whether the recombinant measles virus vaccine strain rMV-Hu191 has an oncolytic effect against ESCC cells in vitro and in vivo and elucidate the underlying mechanisms. Our results showed that rMV-Hu191 could efficiently replicate in and kill ESCC cells through caspase-3/GSDME-mediated pyroptosis. Mechanistically, rMV-Hu191 triggers mitochondrial dysfunction to induce pyroptosis, which is mediated by BAK (BCL2 antagonist/killer 1) or BAX (BCL2 associated X). Further analysis revealed that rMV-Hu191 activates inflammatory signaling in ESCC cells, which may enhance the oncolytic efficiency. Moreover, intratumoral injection of rMV-Hu191 induced dramatic tumor regression in an ESCC xenograft model. Collectively, these findings imply that rMV-Hu191 exhibits an antitumor effect through BAK/BAX-dependent caspase-3/GSDME-mediated pyroptosis and provides a potentially promising new therapy for ESCC treatment.

## Introduction

Esophageal cancer (EC) is among the highest morbidity and mortality worldwide and seriously threatens human health [1]. It was reported that over 540,000 people worldwide died of EC in 2020 [2]. The two main types of EC are esophageal squamous cell carcinoma and esophageal adenocarcinoma (EAC), among which squamous cell carcinoma accounts for over 90% of EC in East Asia [3].

The clinical treatment of EC mainly includes surgery, chemotherapy and radiotherapy. In recent years, target therapy and combination therapy have improved overall survival. However, surgery usually results in a reduced quality of life in EC patients [4]. Furthermore, it has been shown that ESCC and EAC have limited responses to systemic therapy after first-line treatment because of the complicated histology, molecular characteristics and heterogeneity [5]. Although traditional therapies have been improved, EC’s 5-year global survival rate is still very poor [6]. Therefore, there is an urgent need to develop new effective treatments to improve the prognosis of EC. Immunotherapy has greatly improved the survival of patients with melanoma and lung cancer [7, 8], so it is hoped that it can also effectively treat EC. However, there are still some problems with the application of immunotherapy in esophageal cancer. Up to now, only Pembrolizumab has been approved by the U.S. Food and Drug Administration (FDA) for the second-line treatment of PD-L1-positive recurrent locally advanced or metastatic esophageal squamous cell carcinoma [9]. Therefore, developing new therapeutic targets or combining multiple immunotherapies is necessary.

Oncolytic therapy has emerged as a promising new immunotherapy for cancer treatment in recent years. Oncolytic viruses (OVs) mediate antitumor responses by selectively replicating in tumor cells and directly lysing them, which induces the release of immune effectors to activate antitumor immune responses without damaging normal cells [10]. In 2005, the China Food and Drug Administration approved the first OV drug, Oncorine (H101), for the treatment of advanced head and neck cancer [11]. In 2015, the US FDA approved the first oncolytic virotherapy, talimogene laherparepvec (T-VEC, or Imlygic®), to treat patients with metastatic melanoma that cannot be surgically removed [12]. Many clinical trials with OVs are now underway with encouraging data or completed. OVs are gaining more traction because of their outstanding safety profiles even at low doses and promising responses, with evidence of intratumoral viral replication and immune cell infiltrates in colorectal carcinoma [13], glioma [14], melanoma [15], and breast cancer [16].

A variety of oncolytic virus, including adenoviruses [17], herpes simplex virus (HSV) [18], measles virus (MV) [19], Newcastle disease virus (NDV) [20], vaccinia virus [21] and vesicular stomatitis virus (VSV) [22], have undergone clinical trial. Engineered attenuated MV strains are a potential oncolytic platform recently developed for cancer treatment [23, 24]. MV exhibits many advantages as an ideal oncolytic virus, including the excellent safety of the oncolytic vaccine strains which are non-transmissible, selectivity for the tumor, nonpathogenic to normal host tissues, lack of genotoxicity, and general immunity in the population [25, 26]. In addition, it is easy to engineer MVs with the reverse genetic system because of their characteristics. Numbers of preclinical and clinical trials using MV derivatives alone or combined with other cancer treatments are ongoing [27].

Measles virus is a negative-strand enveloped RNA virus belonging to the Paramyxoviridae family, *Morbillivirus* genus. The viral genome has a length of approximately 16 kb and encodes six structural and two nonstructural proteins [25]. The glycoprotein hemagglutinin of MV mediates its binding to the receptors of CD46, nectin-4 and SLAM [28]. In general, wild-type MV infects cells via CD150/SLAM on lymphocytes and nectin-4 on epithelial cells, while MV vaccine strains primarily use CD46 as receptors [29], which is commonly over-expressed on the surface of tumor cells. The Chinese measles vaccine strain Hu191 has been approved and used for many years in China with particularly good safety. Therefore, we constructed rMV-Hu191 using the reverse genetic system, and it showed remarkable safety and immunogenicity [30]. However, whether rMV-Hu191 has an oncolytic effect on ESCC and the mechanism remains unexplained.

Pyroptosis is a form of programmed cell death discovered in recent years, characterized by cell rupture and large bubbles emerging from the plasma membrane, ultimately leading to the release of cellular contents and triggering an immune response [31]. Pyroptosis is mediated by the gasdermin family [32]. In addition, GSDME, also known as DFNA5, can induce pyroptosis through caspase-3-mediated cleavage in vitro and suppress tumor growth by activating antitumor immunity [33–35]. Interestingly, our current study found that rMV-Hu191 treatment stimulates ESCC cell mitochondrial dysfunction and induces BAK/BAX-dependent caspase-3/GSDME-mediated pyroptosis. Overall, our data suggest that rMV-Hu191 exhibits oncolytic effects on ESCC and may be a promising candidate for the treatment of ESCC.

## Results

### rMV-Hu191 exerts a significant oncolytic effect on human ESCC cells

**Fig. 1:**
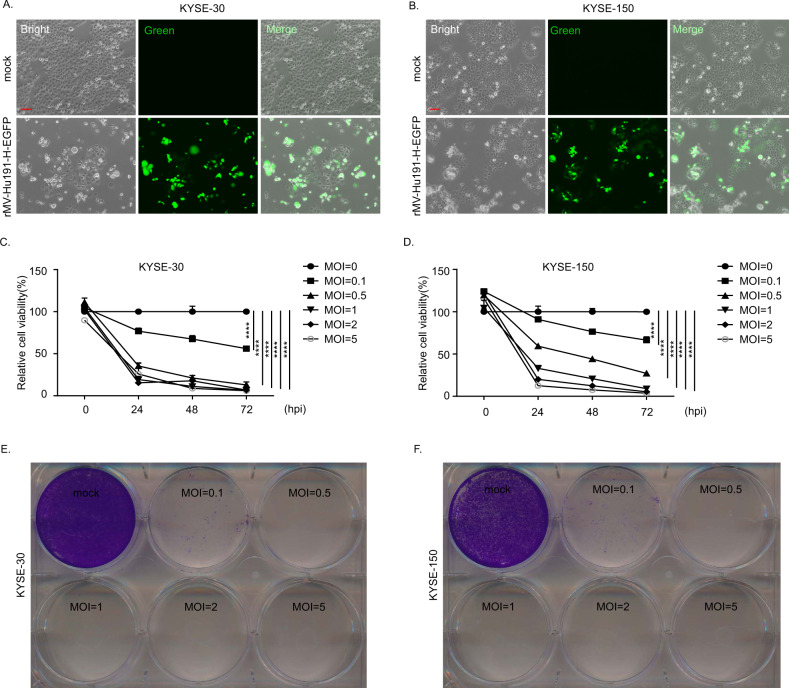
rMV-Hu191 exerts a significant oncolytic effect on human ESCC cells. **A**, **B** Images of KYSE-30 and KYSE-150 cells treated with rMV-Hu191-H-EGFP at an MOI of 0.1 for 48 h. The scale bars represent 100 μm. **C**, **D** KYSE-30 and KYSE-150 cells were treated with rMV-Hu191 at different doses (MOI ranging from 0 to 5) and time courses (0, 24, 48, 72 hpi), and then a CCK8 assay was conducted. The untreated cells were considered as a 100% viability control. MOI multiplicity of infection, hpi hours post-infection. Data are presented as mean ± SEM (*n* = 5). ****p < 0.0001, one-way ANOVA followed by Dunnett’s test. **E**, **F** KYSE-30 and KYSE-150 cells were treated with rMV-Hu191 at different doses (MOI ranging from 0 to 5) for 96 h. Cell-killing efficiency was then determined using crystal staining.

To examine the effect of rMV-Hu191 on ESCC, we treated the ESCC cell lines with rMV-Hu191-H-EGFP and evaluated the infection efficiency by a fluorescence microscope at 48 h after treatment (Fig. 1A, B, Supplementary Fig. S1A). We observed that the ESCC cells were sensitive to rMV-Hu191 infection. Then, we measured the cell viability of ESCC cells using the Cell Counting Kit-8 (CCK8) assay (Fig. 1C, D, Supplementary Fig. S1B). The results showed that treatment with rMV-Hu191 in KYSE-30, KYSE-150 and KYSE-510 cells inhibited cell viability in a dose- and time-dependent manner. To further confirm the cell-killing activity of rMV-Hu191, we conducted crystal violet staining after 96 h post-infection with rMV-Hu191 (Fig. 1E, F, Supplementary Fig. S1C). Consistent with the CCK8 assay, crystal violet staining demonstrated that rMV-Hu191 strongly killed ESCC cells. These results suggest that rMV-Hu191 has a strong oncolytic effect on human ESCC cells. To explore the cytotoxicity of rMV-Hu191 on normal cells, we examined the cell viability of human bronchial epithelial cell line 16HBE after virus treatment. As shown in Supplementary Fig. S1D, only a high MOI of rMV-Hu191 could exert a weaker killing effect on 16HBE cells when compared with ESCC cells.

### rMV-Hu191 induced pyroptosis in ESCC cells

**Fig. 2:**
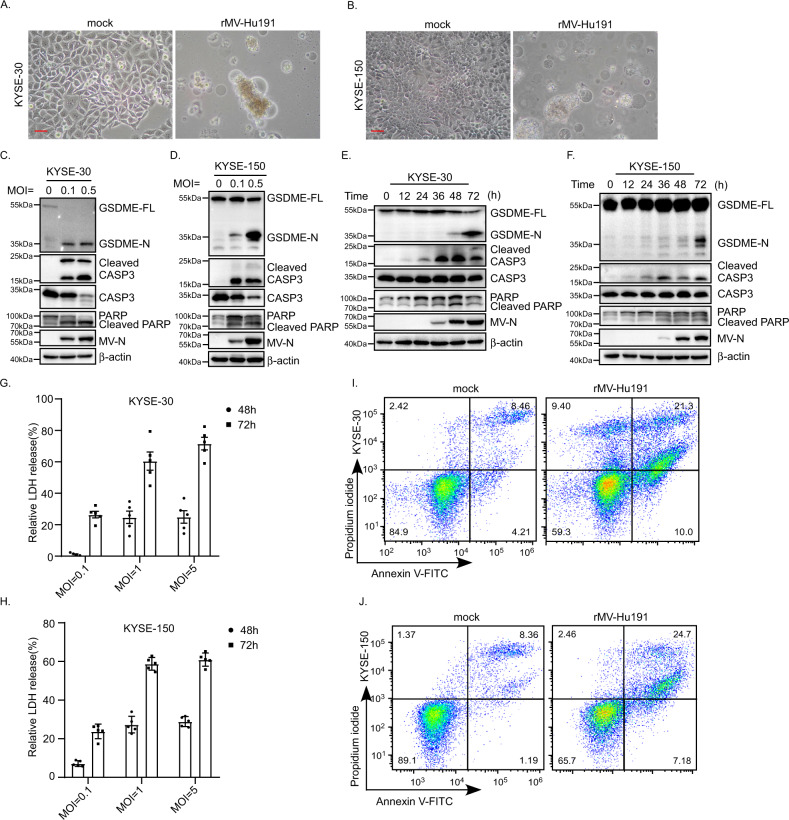
rMV-Hu191 induced pyroptosis in ESCC cells. **A**, **B** KYSE-30 and KYSE-150 cells were treated by rMV-Hu191 at an MOI of 0.1 for 96 h, and then the cell morphology was analyzed by an optical microscope. The scale bars represent 50 μm. **C**, **D** KYSE-30 and KYSE-150 cells were treated by rMV-Hu191 at the indicated MOI for 96 h. Cell lysates were analyzed by immunoblot to detect the cleavage of caspase-3 and GSDME. GSDME-FL full-length GSDME, GSDME-N the N-terminal cleavage product of GSDME. MV-N MV-nucleoprotein. **E**, **F** KYSE-30 and KYSE-150 cells were treated by rMV-Hu191 at an MOI of 0.1 for the indicated time. Cell lysates were analyzed by immunoblot to detect the cleavage of caspase-3 and GSDME. **G**, **H** rMV-Hu191 induced LDH release in KYSE-30 and KYSE-150 cells. Cells were treated by rMV-Hu191 at the indicated doses and time. **I**, **J** KYSE-30 and KYSE-150 cells treated by rMV-Hu191 at an MOI of 0.1 for 48 h were collected and stained with Annexin V-FITC/PI and then subjected to flow cytometry analysis.

Notably, rMV-Hu191-treated ESCC cells exhibited cell swelling with large bubbles from the plasma membrane (Fig. 2A, B, Supplementary Fig. S2A), a typical morphological characteristic of pyroptosis. Recently, pyroptosis was shown to be triggered by the N-terminal domain cleavage of GSDMD and GSDME by caspase-1/-4/-5/-11 and caspase-3, respectively [33, 34, 36]. GSDME was highly expressed in KYSE-30/150/510 cells (Supplementary Fig. S2B). Then we examined the cleavage of GSDME and found that rMV-Hu191 treatment led to elevated levels of the N-terminal cleavage of GSDME in KYSE-30/150/510 cells in a dose- and time-dependent manner (Fig. 2C–F, Supplementary Fig. S2C, D). The nature of cell death was further confirmed by an LDH-release assay and flow cytometry analyses of Annexin V and propidium iodide staining (Fig. 2G–J, Supplementary Fig. S2E, F). Taken together, these results showed that rMV-Hu191 induced the cleavage of GSDME and led to pyroptosis in ESCC cell lines.

### GSDME mediates rMV-Hu191-induced pyroptosis

**Fig. 3:**
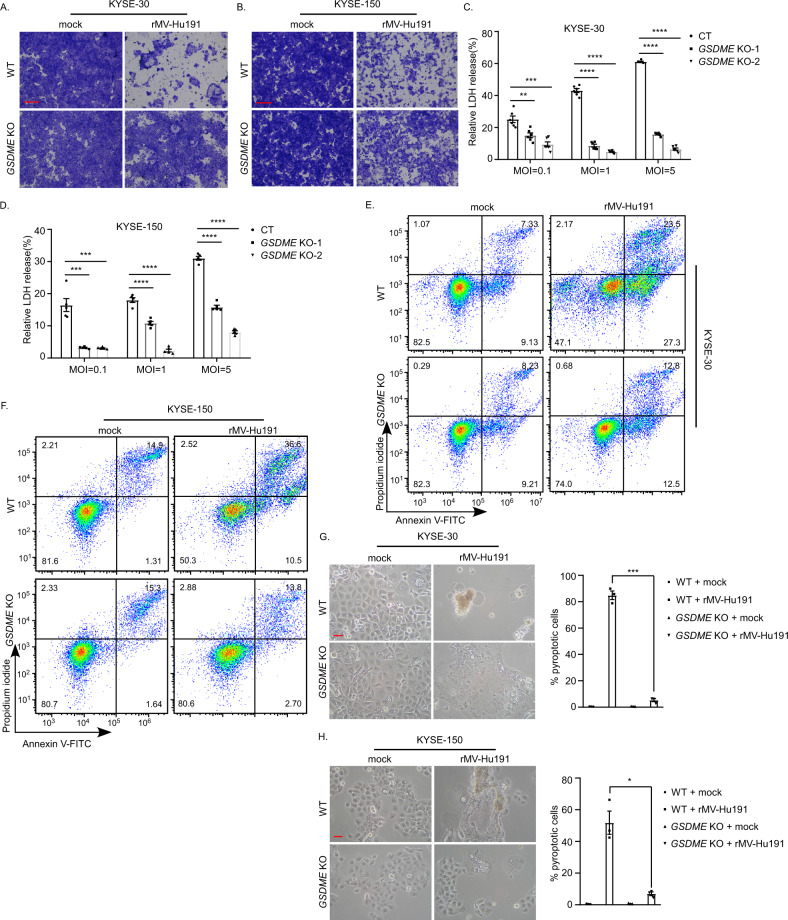
GSDME mediates rMV-Hu191-induced pyroptosis. **A**, **B** KYSE-30 (**A**) or KYSE-150 (**B**) wild-type (WT) and *GSDME* KO cells were treated with rMV-Hu191 at an MOI of 0.1 for 72 h. Cell-killing efficiency was then determined by crystal staining. The scale bars represent 500 μm. **C** LDH release in KYSE-30 WT and *GSDME* KO cells was measured after rMV-Hu191 treatment for 72 h at the indicated doses. Data are presented as mean ± SEM (*n* = 6). **p < 0.01, ***p < 0.001, ****p < 0.0001, one-way ANOVA followed by Dunnett’s test. **D** LDH release in KYSE-150 WT and *GSDME* KO cells was measured after rMV-Hu191 treatment for 72 h at the indicated doses. Data are presented as mean ± SEM (*n* = 5). ***p < 0.001, ****p < 0.0001, one-way ANOVA followed by Dunnett’s test. **E**, **F** Flow cytometry analysis of Annexin V-FITC/PI-stained KYSE-30 (**E**) or KYSE-150 (**F**) WT and *GSDME* KO cells treated by rMV-Hu191 at an MOI of 0.1 for 48 h. **G**, **H** The deficiency of *GSDME* alleviated the pyroptotic morphology of KYSE-30 (**G**) and KYSE-150 (**H**) cells induced by rMV-Hu191. Cells were treated by rMV-Hu191 at an MOI of 0.1 for 72 h. The scale bars represent 50 μm. Pyroptotic cells (cells with large bubbles) were counted from three randomly selected microscopic fields, and the percentage was calculated using the equation pyroptotic cells/total cells × 100%. *p < 0.05, ***p < 0.001, two-tailed Student’s *t*-test.

To investigate the role of GSDME in rMV-Hu191-induced ESCC pyroptosis, two guide RNAs targeting *GSDME* were applied to knock out *GSDME* (Supplementary Fig. S3A, B). Not surprisingly, the deficiency of *GSDME* attenuated the cell-killing efficiency (Fig. 3A, B). The *GSDME* knockout cells displayed lower levels of LDH-release induced by rMV-Hu191 (Fig. 3C, D). Furthermore, the percentage of Annexin V-FITC and/or PI-positive cells decreased in the knockout (KO) group after rMV-Hu191 treatment (Fig. 3E, F). In addition, the proportion of cells with pyroptotic morphology also decreased apparently after *GSDME* knockout (Fig. 3G, H), whereas there was no difference in the expression of the MV-nucleoprotein between WT and *GSDME* KO cells (Supplementary Fig. S3C, D). These observations confirmed the view that GSDME played a key role in rMV-Hu191-induced pyroptosis.

### rMV-Hu191 induces pyroptosis through caspase-3 cleavage of GSDME

**Fig. 4:**
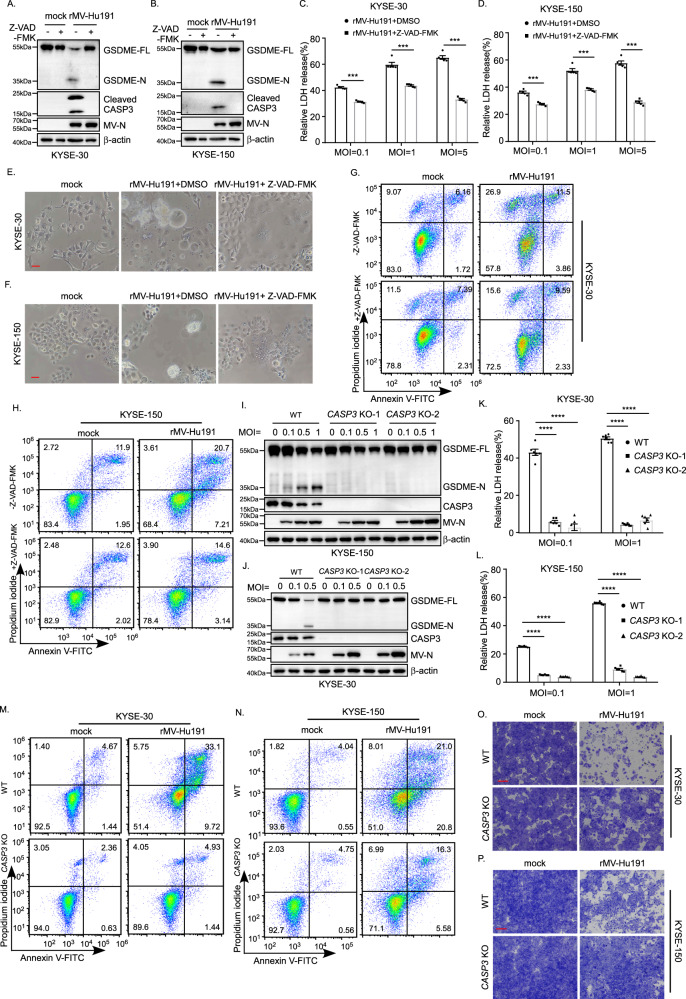
rMV-Hu191 induces pyroptosis through caspase-3 cleavage of GSDME. **A**, **B** The pan-caspase inhibitor Z-VAD-FMK attenuated GSDME cleavage induced by rMV-Hu191 in KYSE-30 (**A**) and KYSE-150 (**B**) cells. Cells were treated by rMV-Hu191 at an MOI of 0.5 with 20 μM Z-VAD-FMK for 48 h and then subjected to immunoblot. **C**, **D** Z-VAD-FMK relieved LDH-release cell death induced by rMV-Hu191 in KYSE-30 (**C**) and KYSE-150 (**D**) cells. LDH release was measured after rMV-Hu191 treatment for 72 h at the indicated doses with 20 μM Z-VAD-FMK. Data are presented as mean ± SEM (*n* = 5). ***p < 0.001, two-tailed Student’s *t*-test. **E**, **F** Z-VAD-FMK alleviated the pyroptosis morphology of KYSE-30 (**E**) and KYSE-150 (**F**) cells induced by rMV-Hu191. Cells were treated by rMV-Hu191 at an MOI of 0.1 for 48 h, and then the cell morphology was analyzed by an optical microscope. The scale bars represent 50 μm. **G**, **H** KYSE-30 and KYSE-150 cells treated by rMV-Hu191 at an MOI of 0.1 with 20 μM Z-VAD-FMK for 48 h were collected and stained with Annexin V-FITC/PI and then subjected to flow cytometry analysis. **I**, **J** Deficiency of caspase-3 attenuated GSDME cleavage induced by rMV-Hu191 in KYSE-30 (**I**) and KYSE-150 (**J**) cells. Cells were treated by rMV-Hu191 for 48 h at the indicated MOI and then subjected to immunoblot. **K**, **L** The LDH release in KYSE-30 (**K**) or KYSE-150 (**L**) WT and *CASP3* KO cells was measured after rMV-Hu191 treatment for 72 h at the indicated doses. Data are presented as mean ± SEM (*n* = 5). ***p < 0.001, one-way ANOVA followed by Dunnett’s test. **M**, **N** KYSE-30 (**M**) or KYSE-150 (**N**) WT and *CASP3* KO cells treated by rMV-Hu191 at an MOI of 0.1 for 48 h were collected and stained with Annexin V-FITC/PI and then subjected to flow cytometry analysis. **O**, **P** KYSE-30 (**O**) or KYSE-150 (**P**) WT and *CASP3* KO cells were treated with rMV-Hu191 at an MOI of 0.1 for 72 h. Cell-killing efficiency was then determined by crystal staining. The scale bars represent 500 μm.

It was reported that activated caspase-3 could cleave GSDME to induce pyroptosis [33]. Therefore, we proposed that caspase-3 is responsible for GSDME cleavage in ESCC cells treated by rMV-Hu191. To confirm this, the pan-caspase inhibitor Z-VAD-FMK was first used (Supplementary Fig. S4A). Interestingly, Z-VAD-FMK not only blocked rMV-Hu191-induced caspase-3 and GSDME cleavage (Fig. 4A, B) but also attenuated the LDH release (Fig. 4C, D). Morphologically, the pyroptosis changes supported the above results (Fig. 4E, F). In addition, the proportion of Annexin V and/or PI-positive cells induced by rMV-Hu191 decreased considerably in the Z-VAD-FMK group compared with the controls (Fig. 4G, H). To further confirm whether caspase-3 is the critical caspase to mediate GSDME cleavage for rMV-Hu191 antitumor activity, we knockout caspase-3 using the CRISPR-Cas9 system in KYSE-30 and KYSE-150 cells (Supplementary Fig. S4B, C). Excitingly, deficiency of caspase-3 blocked the cleavage of GSDME and LDH-release induced by rMV-Hu191 (Fig. 4I–L). Moreover, knockout of caspase-3 dramatically reduced the pyroptosis morphologically and the percentage of Annexin V and/or PI-positive cells induced by rMV-Hu191 (Supplementary Fig. S4D, E, Fig. 4M, N). The attenuated cell-killing activity in the caspase-3 knockout group further verified the view that caspase-3 is required for rMV-Hu191-induced pyroptosis (Fig. 4O, P).

### rMV-Hu191 triggers mitochondrial dysfunction in ESCC cells to induce pyroptosis, which is mediated by BAK or BAX

**Fig. 5:**
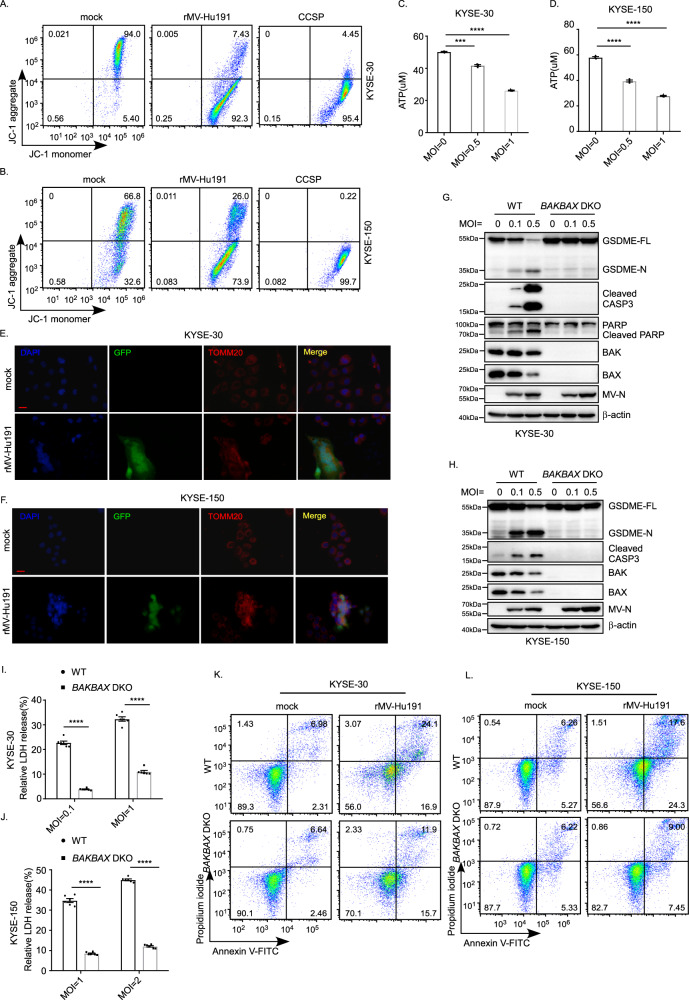
rMV-Hu191 triggers mitochondrial dysfunction in ESCC cells to induce pyroptosis, which is mediated by BAK or BAX. **A**, **B** KYSE-30 (**A**) and KYSE-150 (**B**) cells were treated by rMV-Hu191 at an MOI of 0.5 for 48 h, and the JC-1 assay was then performed to detect mitochondrial membrane potential. **C**, **D** ATP levels in control and rMV-Hu191-treated KYSE-30 (**C**) and KYSE-150 (**D**) cells were evaluated using the ATP assay kit. Cells were treated by rMV-Hu191 for 48 h at the indicated MOI. Data are presented as mean ± SEM (*n* = 3). ***p < 0.001, ****p < 0.0001, one-way ANOVA followed by Dunnett’s test. **E**, **F** Immunofluorescence staining of control and rMV-Hu191-H-EGFP-treated KYSE-30 (**E**) or KYSE-150 (**F**) cells with TOMM20 antibody revealed the disorganization of mitochondria. Cells were treated by rMV-Hu191-H-EGFP for 48 h at an MOI of 0.1. The scale bars represent 50 μm. **G**, **H** Deficiency of *BAK/BAX* attenuated GSDME cleavage induced by rMV-Hu191 in KYSE-30 (**G**) and KYSE-150 (**H**) cells. Cells were treated by rMV-Hu191 for 48 h at the indicated MOI and then subjected to immunoblot. **I**, **J** Loss of *BAK/BAX* relieved LDH-release cell death induced by rMV-Hu191 in KYSE-30 (**I**) and KYSE-150 (**J**) cells. LDH release was measured after rMV-Hu191 treatment for 72 h at the indicated doses. Data are presented as mean ± SEM (*n* = 6). ****p < 0.0001, two-tailed Student’s *t*-test. **K**, **L** KYSE-30 (**K**) or KYSE-150 (**L**) WT and *BAK/BAX* DKO cells treated by rMV-Hu191 at an MOI of 0.1 for 48 h were collected and stained with Annexin V-FITC/PI and then subjected to flow cytometry analysis.

In order to determine the association between mitochondrial function and pyroptosis induction after rMV-Hu191 treatment, the effects of rMV-Hu191 on mitochondrial function were evaluated. We performed a JC-1 assay, and the results showed that rMV-Hu191 decreased the mitochondrial membrane potential in KYSE-30 and KYSE-150 cells (Fig. 5A, B). The ATP levels decreased remarkably after rMV-Hu191 treatment, as detected by the ATP assay kit (Fig. 5C, D). Immunofluorescence analysis of the outer membrane marker TOMM20 (translocase of outer mitochondrial membrane 20) revealed that rMV-Hu191 triggered severe mitochondrial dysfunction (Fig. 5E, F). These results implied that mitochondrial dysfunction might be involved in caspase-3/GSDME-mediated ESCC cell pyroptosis in the presence of rMV-Hu191.

It was reported that chemotherapy-induced pyroptosis is mediated by the BAK/BAX-caspase-3-GSDME pathway in colorectal cancer cells [37]. However, whether rMV-Hu191-induced pyroptosis depends on BAK/BAX is unclear. To identify the relationship, we used wild-type (WT) and BAK/BAX DKO ESCC cells and treated them with rMV-Hu191. Compared with WT cells, pyroptosis in *BAK/BAX* DKO cells was inhibited when exposed to rMV-Hu191, as shown by the decreased GSDME cleavage (Fig. 5G, H), the lower levels of LDH release (Fig. 5I, J), the alleviated pyroptotic morphology (Supplementary Fig. S5A, B), and the reduced percentage of Annexin V-FITC and/or PI-positive cells (Fig. 5K, L). Consistently, the attenuated cell-killing activity in *BAK/BAX* DKO cells also verified the necessity of BAK/BAX in rMV-Hu191-induced pyroptosis (Supplementary Fig. S5C, D). In addition, we also observed diminished cleaved caspase-3 in BAK/BAX DKO KYSE-30 cells and KYSE-150 cells, further supporting the idea that BAK/BAX plays an essential role in rMV-Hu191-induced pyroptosis mediated by caspase-3/GSDME. To further assess the contribution of BAK and BAX in rMV-Hu191-induced pyroptosis, *BAK* and *BAX* were knocked out in KYSE-30 and KYSE-150 cells, respectively, using the CRISPR-Cas9 system. As shown in Supplementary Fig. S5E, F, cleaved GSDME and caspase-3 were blocked remarkably in the absence of either *BAK* or *BAX*. All these observations demonstrate that BAK or BAX are both required for rMV-Hu191-induced pyroptosis.

### rMV-Hu191 activates inflammatory signaling in ESCC cells

**Fig. 6:**
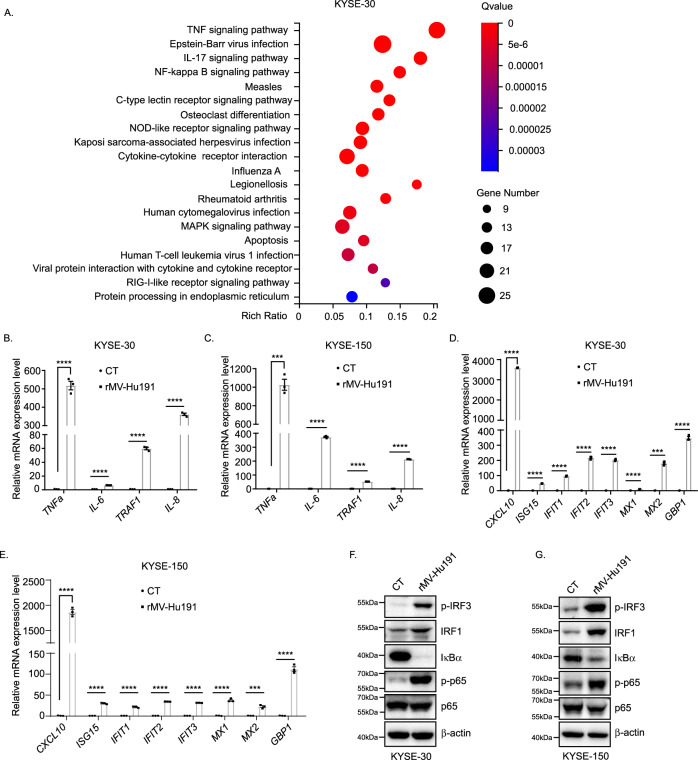
rMV-Hu191 activates inflammatory signaling in ESCC cells. **A** RNA-Seq analysis was used to identify the main pathways that were enriched in differentially expressed genes in rMV-Hu191-treated KYSE-30 cells compared with control cells. Cells were collected for RNA-Seq after being treated by rMV-Hu191 at an MOI of 0.1 for 24 h. **B**–**E**. qRT-PCR analysis of cytokines in control and rMV-Hu191-treated KYSE-30 (**B**, **C**) or KYSE-150 (**D**, **E**) cells. Cells were treated by rMV-Hu191 at an MOI of 0.1 for 24 h. Data are presented as mean ± SEM (*n* = 3). ***p < 0.001, ****p < 0.0001, two-tailed Student’s *t*-test. **F**, **G** Immunoblot revealed activated inflammatory signaling in rMV-Hu191-treated KYSE-30 (**F**) or KYSE-150 (**G**) cells. Cells were treated by rMV-Hu191 at an MOI of 0.1 for 48 h and then subjected to immunoblot.

To further uncover the molecular mechanisms that underlie the oncolytic effect of rMV-Hu191 on ESCC cells, we performed RNA-Seq analysis using RNA isolated from rMV-Hu191-treated and control cells. Among the differentially expressed genes, 189 were upregulated and 7 were downregulated in KYSE-30 cells, and 76 were upregulated and 20 were downregulated in KYSE-150 cells. RNA-Seq data analysis identified a number of pathways enriched in the differentially expressed genes, such as NF-κB, antiviral and apoptosis (Fig. 6A, Supplementary Fig. S6A–C). Importantly, NF-kB signaling was among the most significantly affected pathway. Compared with the controls, the rMV-Hu191-treated cells had increased expression of a battery of inflammation-related genes. It has been reported that the type I IFN signaling pathway and TNF signaling pathway trigger pyroptosis to initiate proinflammatory responses against virus infection [38, 39]. Analysis of mRNA from rMV-Hu191-treated ESCC cell lines revealed that TNF and IFN, two major proinflammatory signaling pathways, were notably activated in the rMV-Hu191-treated ESCC cell lines. qRT-PCR analysis and immunoblot further confirmed this finding (Fig. 6B–G). These data indicate that rMV-Hu191 activates TNF and interferon signaling and promotes the production of proinflammatory cytokines, which may enhance the oncolytic effect in turn.

### rMV-Hu191 induced tumor regression in an ESCC xenograft model

**Fig. 7:**
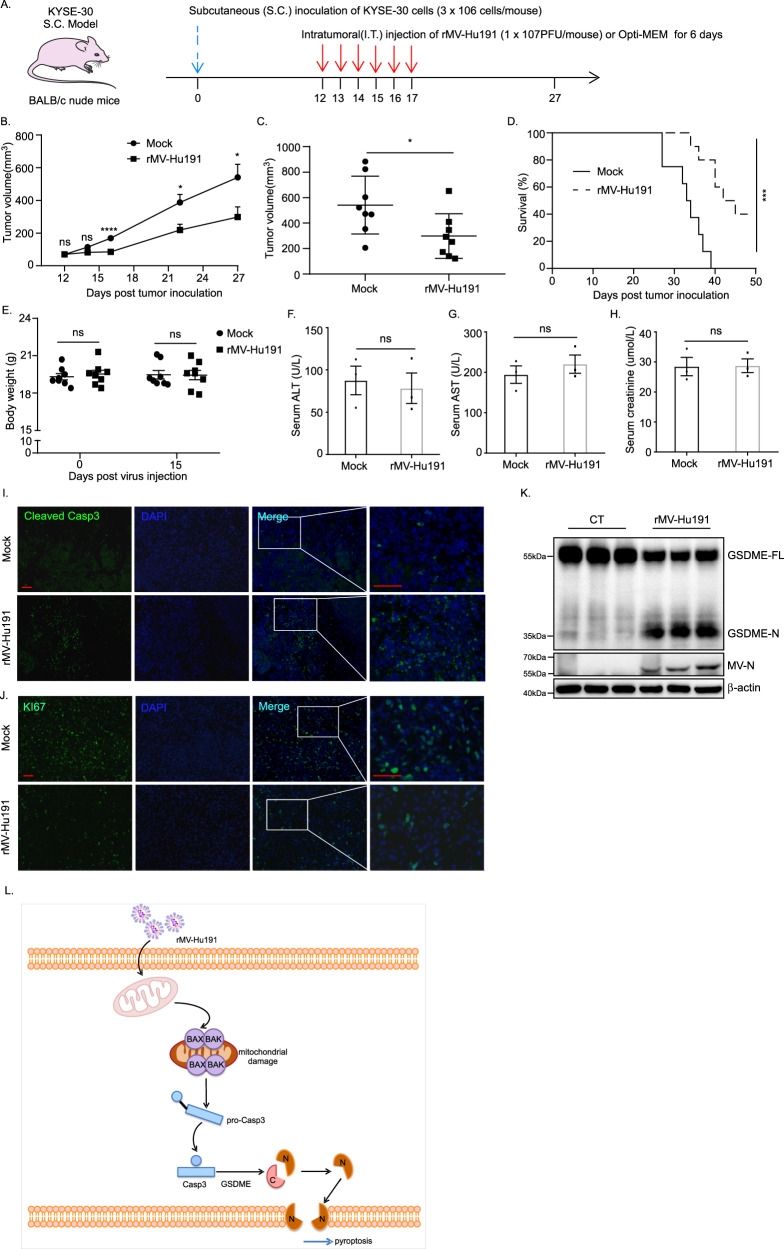
rMV-Hu191 induced tumor regression in an ESCC xenograft model. **A** 3 × 10^6^ KYSE-30 cells were subcutaneously inoculated into BALB/c nude mice to establish the S.C. tumor model, and then 1 × 10^7^ PFU of rMV-Hu191 were administrated intratumorally (I.T.) from day 12 to day 17. **B** Tumors were measured in the long and short dimensions using a vernier caliper, and tumor volumes were estimated using the equation: V = (length × Width^2^)/2. *n* = 8 tumors for each group. *p < 0.05, ****p < 0.0001, two-tailed Student’s *t*-test. The solid line represents the average volume ± SEM. **C** Difference in tumor volume on day 27 post tumor cell inoculation. *p < 0.05, two-tailed Student’s *t*-test. The solid line represents the average volume ± SEM. **D** The Kaplan–Meier survival curves of mice from rMV-Hu191 and mock-treated groups. A log-rank (Mantel–Cox) test was used to analyze the significance of differences between groups. ***p < 0.0001. **E** Body weights of the mock and rMV-Hu191-treated mice were measured on day 0 and 15 post virus injection. The solid line represents the average weight ± SEM (*n* = 8). ns, no significant differences, two-tailed Student’s *t*-test. **F**–**H** The levels of ALT, AST and CR in mouse serum were measured. Data are presented as mean ± SEM (*n* = 3). ns, no significant differences, two-tailed Student’s *t*-test. **I**, **J** Immunofluorescence staining of the mock and rMV-Hu191-treated tumors with the indicated antibodies. The scale bars represent 50 μm. **K** Immunoblot analysis revealed elevated GSDME cleavage in rMV-Hu191-treated tumors. **L** Schematic model for the regulation of pyroptosis in ESCC by rMV-Hu191. rMV-Hu191 exhibits an antitumor effect through BAK/BAX-dependent caspase-3/GSDME-mediated pyroptosis.

To explore the oncolytic effect of rMV-Hu191 in ESCC in vivo, we established an ESCC xenograft model by injection of KYSE-30 cells subcutaneously into the right flank of immunodeficient BALB/c nude mice (Fig. 7A). As expected, intratumoral injection of rMV-Hu191 led to a significant delay in tumor growth compared with the mock group (Fig. 7B, C). Meanwhile, rMV-Hu191 administration increased the survival rate of the mice (Fig. 7D). Furthermore, we examined the mice’s body weights to evaluate the general toxicity of rMV-Hu191 in vivo and found no significant difference between the two groups (Fig. 7E). Moreover, no significant difference in serum ALT (alanine transaminase), AST (aspartate aminotransferase) and CR (creatinine) levels in the two groups of mice was observed (Fig. 7F–H), indicating a good tolerance of the animals to rMV-Hu191. Immunofluorescence staining of tumor tissue showed remarkably elevated cleaved caspase-3 and reduced Ki-67 expression in the treated group compared with the mock group (Fig. 7I, J), demonstrating apoptosis occurred in rMV-Hu191-treated tumors. Consistent with the results in ESCC cell lines, rMV-Hu191 treatment induced GSDME cleavage in the xenograft tumor tissues (Fig. 7K). Taken together, these data suggest that rMV-Hu191 exerts an oncolytic effect on ESCC by disrupting tumor cell growth and inducing programmed cell death.

## Discussion

Oncolytic MV has recently been proven as an effective and promising cancer therapeutic strategy, and several clinical trials for cancer treatment are ongoing. However, there is limited data about the oncolytic measles virotherapy in ESCC. As mentioned above, we have successfully established an efficient reverse genetics system for the Chinese measles vaccine MV-Hu191 which showed remarkable safety and immunogenicity [30]. In this study, we explored the oncolytic effect of rMV-Hu191 on human ESCC cells in vitro and in vivo. We found that rMV-Hu191 induced pyroptotic morphologies, increased the proportion of Annexin V and/or PI-positive cells, elevated levels of LDH release and GSDME cleavage, demonstrating that pyroptosis was induced by rMV-Hu191 in ESCC cells. In addition, loss of *GSDME* inhibited pyroptotic morphology, LDH release, GSDME cleavage and the oncolytic activity, suggesting that rMV-Hu191 plays an oncolytic effect through GSDME-mediated pyroptosis.

It is well known that GSDME is cleaved and activated specifically by caspase-3 in chemotherapy drug-induced pyroptosis [33]. The present study shows that the pan-caspase inhibitor Z-VAD-FMK eliminates pyroptosis and blocks the oncolytic effect induced by rMV-Hu191. Furthermore, caspase-3 knockout blocked both GSDME cleavage and pyroptosis, indicating caspase-3 was essential to rMV-Hu191-induced pyroptosis.

Normal mitochondrial function is necessary to maintain homeostasis in the body. Multiple death signals stimulate mitochondria and trigger the homo-oligomerization of proapoptotic family members BAK or BAX, resulting in the critical initiating event for mitochondrial dysfunction [40, 41]. Meanwhile, BAK and BAX promote cell death by inducing mitochondrial outer membrane permeabilization, which will cause activation of the caspase cascade [42]. It is important to note that severe mitochondrial damage occurred during rMV-Hu191 treatment, as shown by decreased mitochondrial membrane potential in ESCC cells. Therefore, we established *BAK/BAX* DKO ESCC cells to further investigate the role of BAK and BAX in rMV-Hu191-induced pyroptosis. Remarkably, *BAK/BAX* DKO attenuated rMV-Hu191-induced GSDME cleavage, LDH release, pyroptotic morphology and oncolytic effect apparently, suggesting that BAK and BAX contributed to the activation of the caspase-3/GSDME cascade and subsequent pyroptosis pathways.

Despite the obvious safety of MV, the challenge arising from oncolytic measles virotherapy is the antiviral response in the general population which may hamper overall oncolytic efficacy due to premature viral clearance [43]. Therefore, various strategies have been developed to evade antiviral immunity. It has been reported that replacing the P/V/C, N and L genes of the WT strain with attenuated oncolytic strain can circumvent the cellular IFN response and increase viral titers, resulting in cell-killing incompetent interferon cells [44]. rMV-Hu191 used in this study was established by altering the S-adenosylmethionine (SAM) binding site in the large polymerase (L) protein of the Chinese MV vaccine Hu191 to improve safety and immunogenicity. The engineered rMV-Hu191 was more attenuated in vitro and in vivo than the parental strain and triggered higher neutralizing antibody levels than the parental strain [30]. Furthermore, our RNA-Seq analysis uncovered a significant number of inflammatory pathways activated in rMV-Hu191-treated ESCC cells. Interestingly, the cell-killing effect was still remarkable, possibly due to the severe mitochondrial damage induced by the inflammatory response. These studies imply that a good balance of antitumor immunity and antiviral immunity would improve the effect of oncolytic virotherapy, which will be a major research direction in the future.

MV is a primate-adapted virus that specifically targets cells expressing the human CD46 receptor. Due to the absence of the human CD46 receptor in mouse cells, they are not susceptible to the virus, making MV well tolerated in mice [45]. Therefore, a subcutaneous transplanted tumor model of human ESCC in nude mice was used in this study to disclose the role of rMV-Hu191 in vivo. Consistent with our in vitro data, intratumoral injection of rMV-Hu191 strongly dampened tumor growth in nude mice without obvious toxicity observed in body weight and physiological function. Collectively, our current study indicates that rMV-Hu191 will be a promising candidate for oncolytic virotherapy of human ESCC, and next, we will try to develop an improved mouse model for preclinical development.

In summary, we elucidate the antitumor effect of rMV-Hu191 on ESCC cells, which is induced by caspase-3 activation and GSMDE cleavage-mediated pyroptosis. Furthermore, identifying BAK and BAX as upstream effectors of caspase-3 activation in rMV-Hu191 treatment by inducing mitochondrial injury also provides new targets to modulate pyroptosis. Moreover, exploring the role of pyroptosis in the pathogenesis of human diseases may provide new ideas and effective therapeutic targets for disease prevention and treatment. Overall, our results suggest a possibility of MV in esophagus cancer treatment, and future studies should focus on further virus engineering and delivery to improve selectivity and clinical outcomes.

## Materials and methods

### Cell lines and culture

The human ESCC cell lines KYSE-30, KYSE-150 and KYSE-510 were from the JCRB cell bank (Japanese Collection of Research Bioresources cell bank) and cultured in RMPI 1640 (GIBCO) supplemented with 10% fetal bovine serum (FBS, GIBCO) and 1% penicillin/streptomycin (GIBCO) at 37 °C with 5% CO_2_. Vero cells (ATCC-CCL-81) were cultured in DMEM (GIBCO) supplemented with 10% fetal bovine serum (FBS, GIBCO) and 1% penicillin/streptomycin (GIBCO) at 37 °C with 5% CO_2_.

### Virus construction, titration and infection

The construction, propagation and purification of the recombinant MV vaccine strain (rMV-Hu191) were performed as previously described [30]. For virus titration detection, Vero cells were used in plaque assays to quantify the infectious titers of rMV-Hu191. For virus infection assays, cells were washed with PBS twice and then incubated with rMV-Hu191 at a certain MOI in an Opti-MEM medium. After incubation at 37 °C for 1 h, the medium containing the virus was replaced with a fresh medium.

### CRISPR/Cas9 knockout cells

*GSDME* gRNAs (5′-TAAGTTACAGCTTCTAAGTC-3′ and 5′-CAGTTTTTATCCCTCACCCT-3′), *CASP3* gRNAs (5′-GTGGAATTGATGCGTGATGTT-3′ and 5′-CCGAAAGGTGGCAACAGAATT-3′), *BAX* gRNAs (5′-TCGGAAAAAGACCTCTCGGG-3′ and 5′-GTTCCGGCACCTTGGTGCAC-3′) and *BAK* gRNAs (5′-CATGAAGTCGACCACGAAGC-3′ and 5′-GCATGAAGTCGACCACGAAG-3′) were cloned into LentiCRISPR v2 (Addgene). Following transfection and transient selection with puromycin for 2 days, cells were cultured without puromycin. Knockout clones were selected through immunoblot analysis. Two independent clones were analyzed, and the parental WT cells were used as a control.

### Antibodies and reagents

Antibodies for GSDME (ab215191) and Measles nucleoprotein (ab106292) were purchased from Abcam. Antibodies for caspase-3 (#9662), cleaved caspase-3 (#9579), BAX (#2772), BAK (#12105) and PARP (#9532) were purchased from Cell Signaling Technology. Antibody for β-actin (AC038) was purchased from ABclonal Technology. Antibody for TOMM20 (66777-1-Ig) was purchased from Proteintech Group, Inc. Antibody for Ki-67 was obtained from BD Biosciences (550609). Opti-MEM was sourced from Gibco.

### Immunoblot

Cells were lysed in 1× SDS loading buffer (Beyotime, P0015L) and boiled at 100 °C for 10 min. The lysates were separated by SDS-PAGE and transferred to PVDF membranes (Millipore), which were then blocked with 5% milk in TBST buffer (150 mM NaCl, 20 mM Tris-HCl pH 7.6, 0.1% Tween-20) for 1 h at room temperature (RT) before incubation with primary antibodies overnight at 4 °C. After washing with TBST three times, membranes were incubated with a secondary antibody for 1 h at RT and then washed three times with TBST before adding the FDbio-Femto Ecl (Hangzhou Fdbio science, FD8030) for detection. Immunoblot images were captured by ChemiScope5600 (Clinx, Shanghai).

### Immunofluorescence and immunohistochemistry

For immunofluorescence, cells were seeded on round glass coverslips and subjected to the indicated treatment. Cells were washed with PBS twice and fixed with 4% PFA for 20 min at RT, followed by permeabilization with 0.5% TritonX-100/PBS for 5 min at RT. The cells were then incubated with primary antibodies overnight at 4 °C after being blocked with 3% BSA/PBS for 1 h at RT and followed by incubation with Alexa Fluor-labeled secondary antibodies for 1 h at RT. Nuclei were stained with DAPI.

For tissue immunofluorescence staining, tumors were fixed with 4% paraformaldehyde (PFA) for 4 h at 4 °C. After dehydration and processing, the tumors were embedded in paraffin for sectioning and immunofluorescence. All tissues were sectioned at 6 μm thickness for histological analysis. EDTA Antigen Retrieval Solution (Beyotime, P0085) was used for antigen retrieval. Primary antibodies were incubated overnight at 4 °C after being blocked with 3% BSA/PBS for 1 h at RT. Alexa Fluor-labeled secondary antibodies were used. Nuclei were stained with DAPI.

### Reverse transcription (RT), quantitative PCR (qPCR) and RNA-Seq analysis

Total RNA was extracted with RNAiso Plus reagent (Takara), and cDNA was generated using PrimeScript™ RT Master Mix (Takara) according to the manufacturer’s protocol. The cDNA was subjected to quantification with AceQ Universal SYBR qPCR Master Mix (Vazyme). The 2^−ΔΔCT^ method has been extensively used as a relative quantification strategy for quantitative real-time polymerase chain reaction (qPCR) data analysis. Relative quantification was expressed as 2^−ΔCt^, where ΔCt is the difference between the main Ct value of triplicates of the sample and that of GAPDH control. Quantitative PCR primers are listed in Supplementary Table 1. For RNA-seq, samples were analyzed by BGI-Shenzhen, China. The dataset was selected from differentially expressed genes with a cutoff of at least a twofold change in expression level (*p*-value < 0.05). Pathway enrichment analysis and network analysis were performed on differentially expressed genes using Ingenuity Pathway Analysis.

### Cell viability assays and cell-death assays

Human ESCC cells were treated with rMV-Hu191 at the indicated time and MOI and then subjected to microscopy imaging, cell viability/death, immunoblotting analyses, or flow cytometry. Cell viability was detected using the CCK-8 assay (Targetmol, C0005). LDH release was measured to analyze cell death using the cytotoxicity assay kit (Beyotime, C0016) according to the manufacturer’s instructions. ATP levels were measured using the ATP assay kit (Beyotime, S0026). For flow cytometry analyses, Cells were collected, washed with PBS twice and stained using the Annexin V-FITC/PI Apoptosis Assay Kit (Meilunbio, MA0220) according to the manufacturer’s instructions. Stained cells were analyzed with a Beckman CytoFlex, and data were processed using FlowJo software.

### Nude mouse tumor model

BALB/c nude mice were purchased from the Zhejiang Academy of Medical Sciences. 5 weeks-old female mice were used. No statistical method was used to predetermine sample size in the animal studies. Animal experiments were conducted in accordance with the ARRIVE guidelines and approved by the Animal Care and Use Committee of Zhejiang Chinese Medical University. A total of 3 × 10^6^ KYSE-30 cells were subcutaneously inoculated into the right-back flanks of nude mice. Mice were observed regularly for tumor presence by visual inspection. Tumors were measured in the long and short dimensions, and tumor volumes were estimated using the equation: V = (length × Width^2^)/2.

On post-implantation day 12, mice were randomly divided into two groups when the average volume reached 70 mm^3^. Mice from the rMV-Hu191-treated group received intratumoral administration of 1 × 10^7^ PFU of virus suspension in 100 μl Opti-MEM from days 12 to 17 post-implantation, while mice from the mock-treated group were injected with an equal volume of Opti-MEM. On post-implantation day 20, three mice from each group were sacrificed to collect tumor tissues for immunoblot and tissue immunofluorescence staining. Serum ALT, AST and CR were measured using commercial kits (Nanjing Jiancheng Bioengineering Institute, China). The other eight mice in each group were housed until the day of euthanasia. Tumor-bearing mice were euthanized when the tumor exceeded 15 mm in either dimension, festered or the animal body weight loss exceeded 20%.

### Statistical analysis

Statistical analyses were performed with a two-tailed, unpaired Student’s *t*-test. Multiple group comparisons were performed using one-way ANOVA followed by Dunnett’s test. Comparisons between multiple groups with different treatments were performed using two-way ANOVA followed by Dunnett’s test. All analyses were performed by GraphPad Prism 6.0. P-values < 0.05 were considered to be significant. Quantitative data were presented as mean ± SEM. No statistical methods were used to predetermine the sample. The experiments were not randomized, and the investigators were not blinded to allocation during experiments and outcome assessment.

## Supplementary information

supplementary figure legends

supplementary figures-R

Original Data File

Original Data File

## Data Availability

The datasets presented in this study can be found in online repositories. The names of the repositories and accession numbers can be found below: https://www.ncbi.nlm.nih.gov/sra/PRJNA858483. All other remaining data are included in the article and supplementary information files or available from the authors upon reasonable request.
