# Supplementary material for: Recombinant measles virus vaccine rMV-Hu191 exerts an oncolytic effect on esophageal squamous cell carcinoma via caspase-3/GSDME-mediated pyroptosis
Source: Cell Death Discov. 2023 May 19;9:171. doi: 10.1038/s41420-023-01466-2 (PMC10195838; doi:10.1038/s41420-023-01466-2)
Supplement: Supplementary file 1 — supplementary figure legends [file 41420_2023_1466_MOESM1_ESM.docx]

**Supplementary table legends:**

**Table 1: Quantitative PCR primers sequence.**

**Supplementary figure legends:**

**Fig S1. rMV-Hu191 exerts a significant oncolytic effect on human ESCC cells.** (A). Images of KYSE-510 cells treated with rMV-Hu191-H-EGFP at an MOI of 0.1 for 48 h. The scale bars represent 100 μm. (B). KYSE-510 cells were treated with rMV-Hu191 at different doses (MOI ranging from 0 to 5) and time course (0, 24, 48, 72 hpi). Cell viability was then determined by CCK8 assay. The untreated cells were considered as a 100% viability control. Data are presented as mean ± SEM (n = 5). **** P < 0.0001, one-way ANOVA followed by Dunnett’s test. (C). KYSE-510 cells were treated with rMV-Hu191 at different doses (MOI ranging from 0 to 5) for 96 h. Cell-killing efficiency was then determined using crystal staining. (D). 16HBE cells were treated with rMV-Hu191 at different doses (MOI ranging from 0 to 5) and time course (0, 24, 48, 72 hpi). Cell viability was then determined by CCK8 assay. The untreated cells were considered as a 100% viability control. Data are presented as mean ± SEM (n = 5). *** p < 0.001, **** p < 0.0001, one-way ANOVA followed by Dunnett’s test.

**Fig S2. rMV-Hu191 induced pyroptosis in ESCC cells.** (A). KYSE-510 cells were treated by rMV-Hu191 at an MOI of 0.1 for 96 h and then the cell morphology was analyzed by an optical microscope. The scale bars represent 50μm. (B). Immunoblot to detect the expression of GSDME in various cell lines. (C). KYSE-510 cells were treated by rMV-Hu191 at the indicated MOI for 72 h. Cell lysates were analyzed by immunoblot to detect the cleavage of caspase-3 and GSDME. (D). KYSE-510 cells were treated by rMV-Hu191 at an MOI of 0.5 for the indicated time. Cell lysates were analyzed by immunoblot to detect the cleavage of caspase-3 and GSDME. (E). rMV-Hu191 induced LDH release in KYSE-510 cells. Cells were treated by rMV-Hu191 at the indicated doses and time. (F). KYSE-510 cells treated by rMV-Hu191 at an MOI of 0.5 for 48 h were collected and stained with Annexin V-FITC/PI and then subjected to flow cytometry analysis.

**Fig S3. GSDME mediates rMV-Hu191-induced pyroptosis**. (A-B). The deficiency of *GSDME* in KYSE-30 (A) and KYSE-150 (B) cells was detected by the immunoblot. (C-D). Immunoblot to detect the MV-nucleoprotein expression in KYSE-30 (C) or KYSE-150 (D) WT and *GSDME* KO cells.

**Fig S4. rMV-Hu191 induces pyroptosis through caspase-3 cleavage of GSDME.** (A). Z-VAD-FMK attenuated cell-killing activity induced by rMV-Hu191 in KYSE-150 cells. Cells were treated by rMV-Hu191 at an MOI of 0.1 for 72 h and the cell-killing efficiency was then determined by crystal staining. Fer-1: Ferrostatin-1, ferroptosis inhibitor; Nec-1: Necrostatin-1, necroptosis inhibitor. The scale bars represent 500 μm. (B-C). Immunoblot was used to detect the knockout efficiency of caspase-3 in KYSE-30 (B) and KYSE-150 (C) cells. (D-E). Deficiency of caspase-3 attenuated the pyroptosis morphology of KYSE-30 (D) and KYSE-150 (E) cells induced by rMV-Hu191. Cells were treated by rMV-Hu191 at an MOI of 0.1 for 72 h and then the cell morphology was analyzed by an optical microscope. The scale bars represent 50μm.

**Fig S5. rMV-Hu191 triggers mitochondrial dysfunction in ESCC cells to induce pyroptosis, which is mediated by BAK or BAX.** (A-B). Deficiency of *BAK/BAX* alleviated pyroptosis morphology of KYSE-30 (A) and KYSE-150 (B) cells induced by rMV-Hu191. Cells were treated by rMV-Hu191 at an MOI of 0.1 for 72 h and then the cell morphology was analyzed by an optical microscope. The scale bars represent 50μm. (C-D). KYSE-30 (C) or KYSE-150 (D) WT and *BAK/BAX* DKO cells were treated with rMV-Hu191 at an MOI of 0.1 for 72 h. Cell-killing activity was then determined by crystal staining. The scale bars represent 500 μm. (E-F). The deficiency of *BAK* or *BAX* alone in KYSE-30 (E) and KYSE-150 (F) cells blocked the cleavage of caspase-3 and GSDME induced by rMV-Hu191. Cells were treated by rMV-Hu191 for 48 h at the indicated MOI and then subjected to immunoblot.

**Fig S6. rMV-Hu191 activates inflammatory signaling in ESCC cells.** (A). Heat map analysis of the highly up-regulated (red) and down-regulated (blue) genes in rMV-Hu191-treated KYSE-30 cells compared with controls. (B). RNA-Seq analysis identified the main pathways that were enriched in differentially expressed genes in rMV-Hu191-treated KYSE-150 cells compared with control cells. Cells were collected for RNA-Seq after being treated by rMV-Hu191 at an MOI of 0.1 for 24 h. (B). Heat map analysis of the highly up-regulated (red) and down-regulated (blue) genes in rMV-Hu191-treated KYSE-150 cells compared with controls.
