# Supplementary figures and images for: Recombinant measles virus vaccine rMV-Hu191 exerts an oncolytic effect on esophageal squamous cell carcinoma via caspase-3/GSDME-mediated pyroptosis
Source: Cell Death Discov. 2023 May 19;9:171. doi: 10.1038/s41420-023-01466-2 (PMC10195838; doi:10.1038/s41420-023-01466-2)

Fig S1

A.

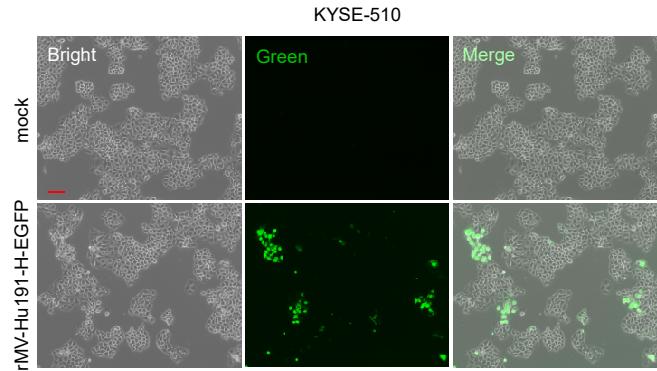

B.

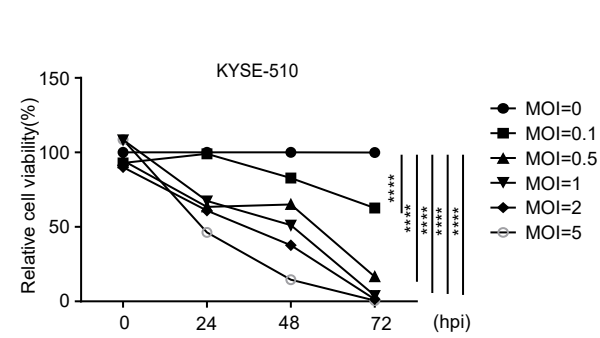

C.

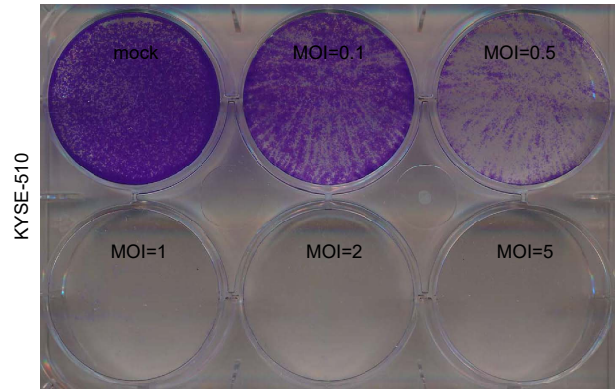

D.

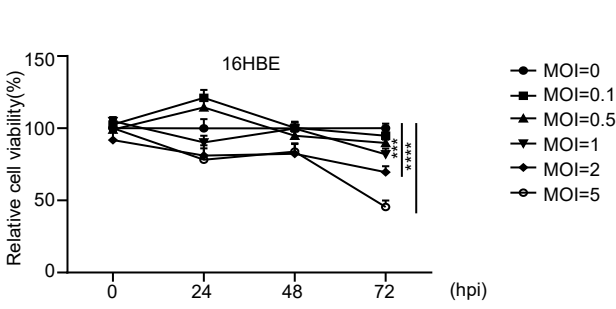

Fig S2

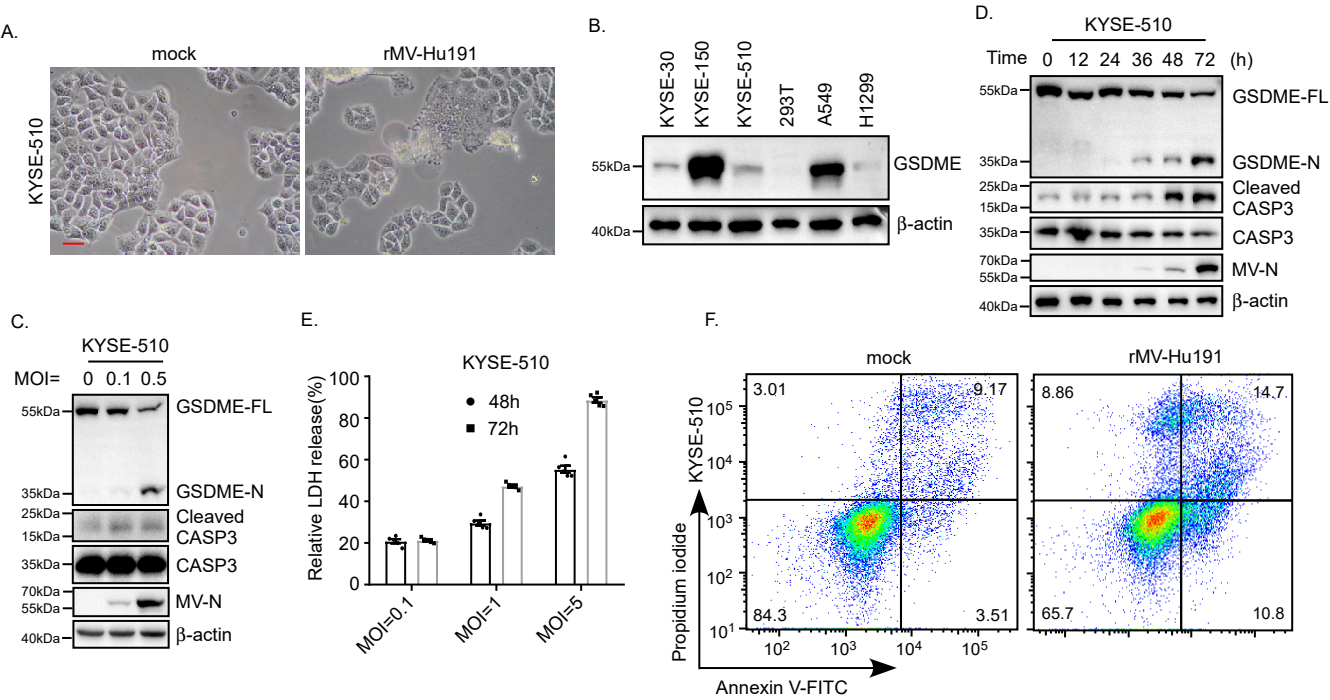

Fig S3

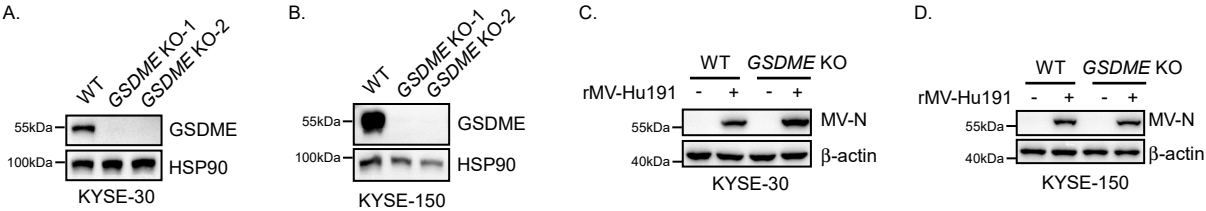

Fig S4

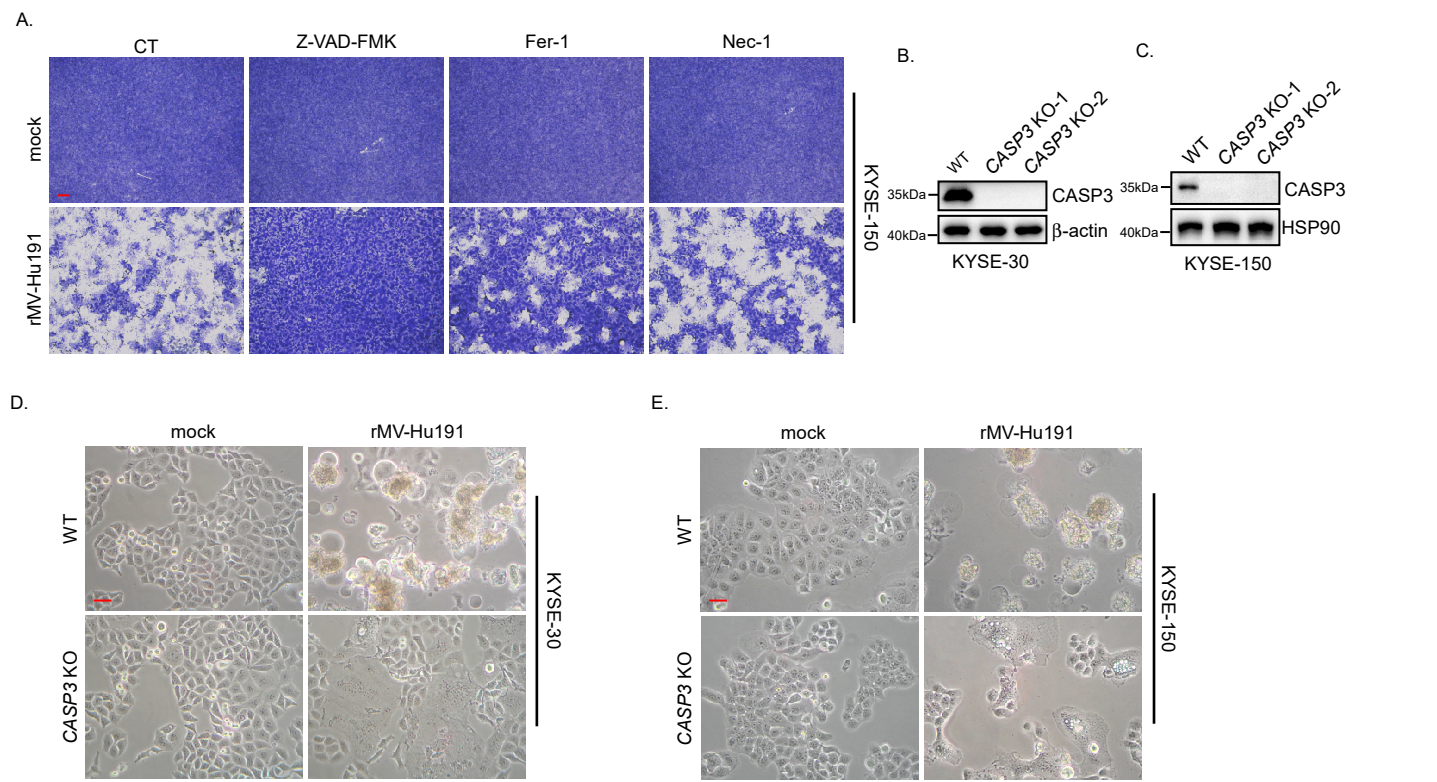

Fig S5

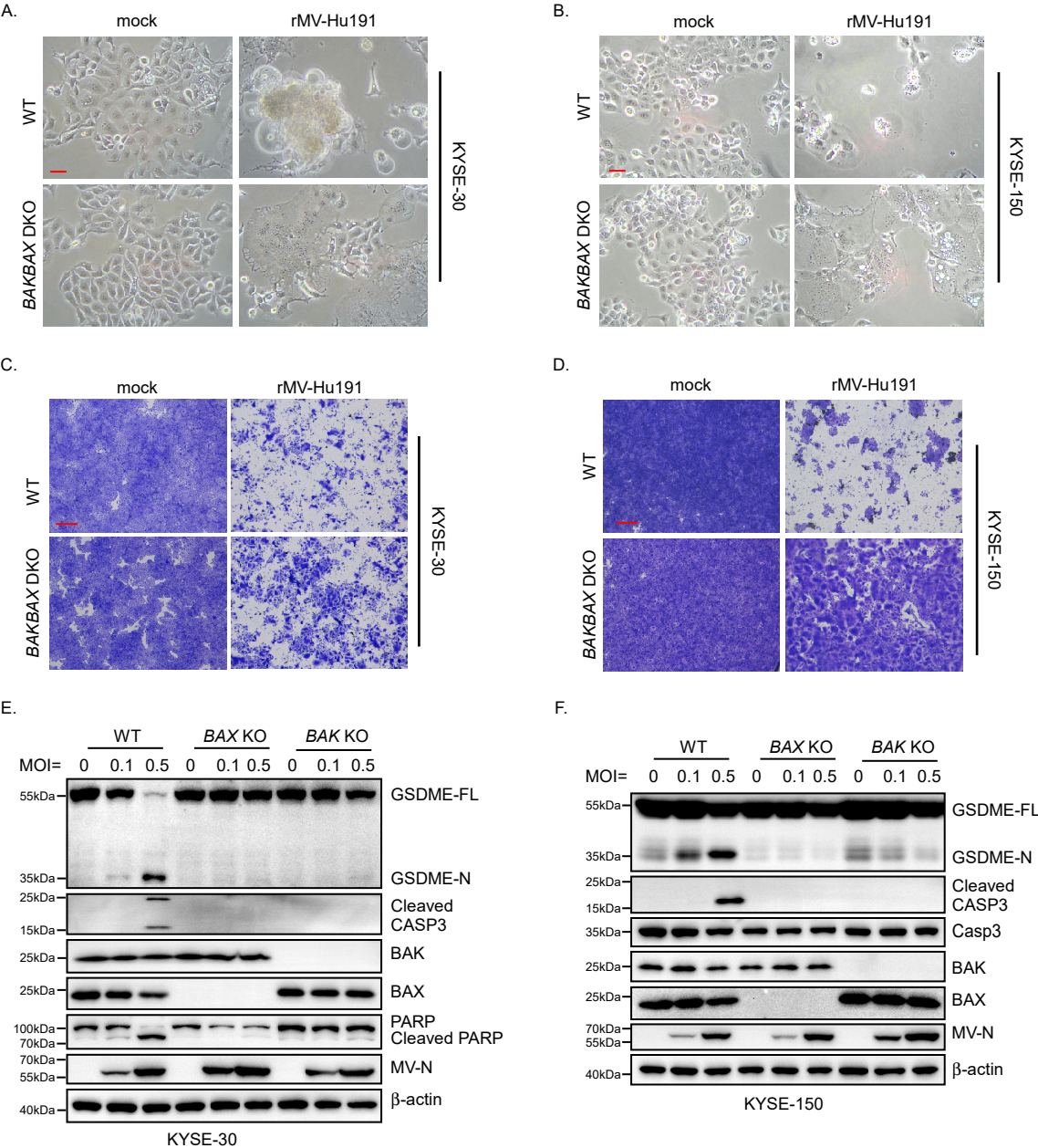

Fig S6

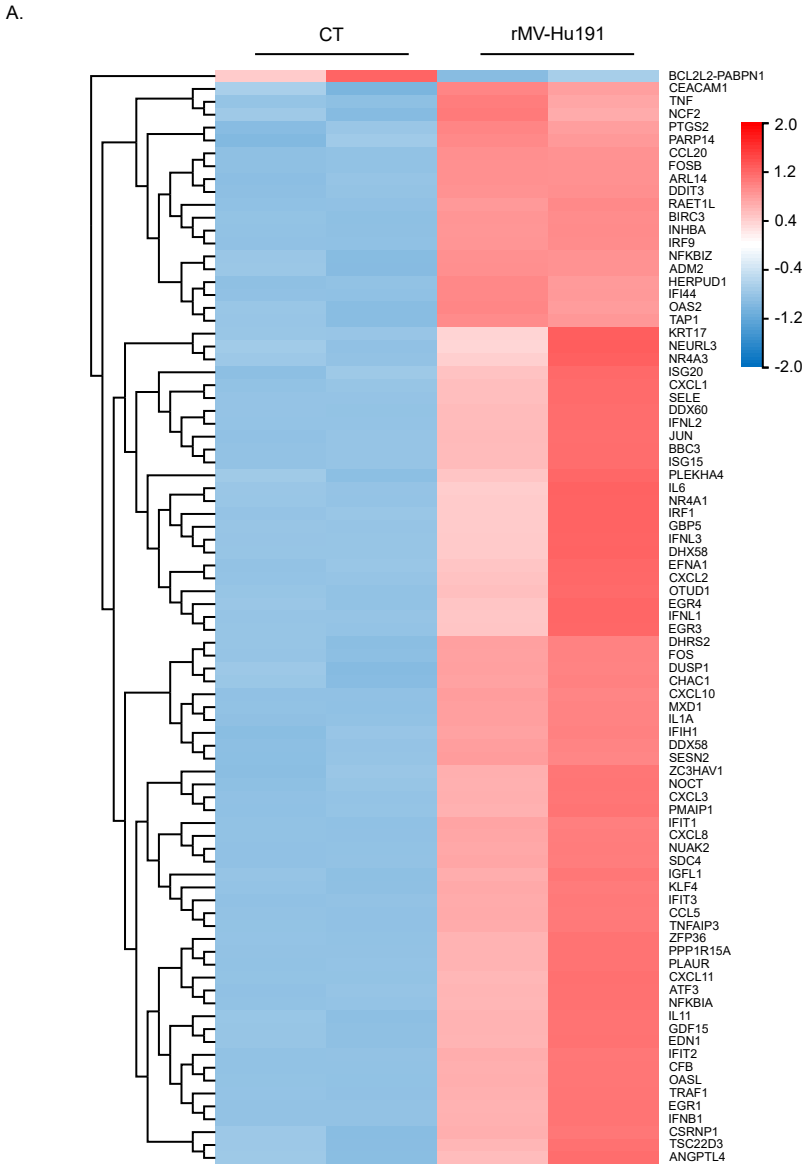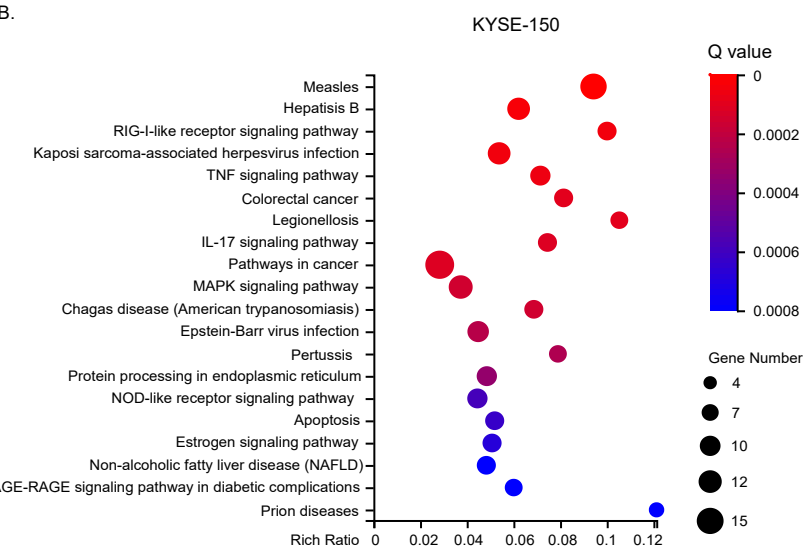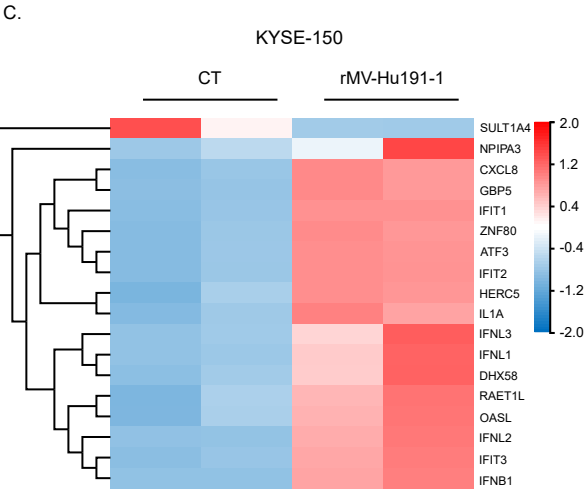

Supplement: Supplementary file 2 — supplementary figures-R [file 41420_2023_1466_MOESM2_ESM.pdf]

Fig 2

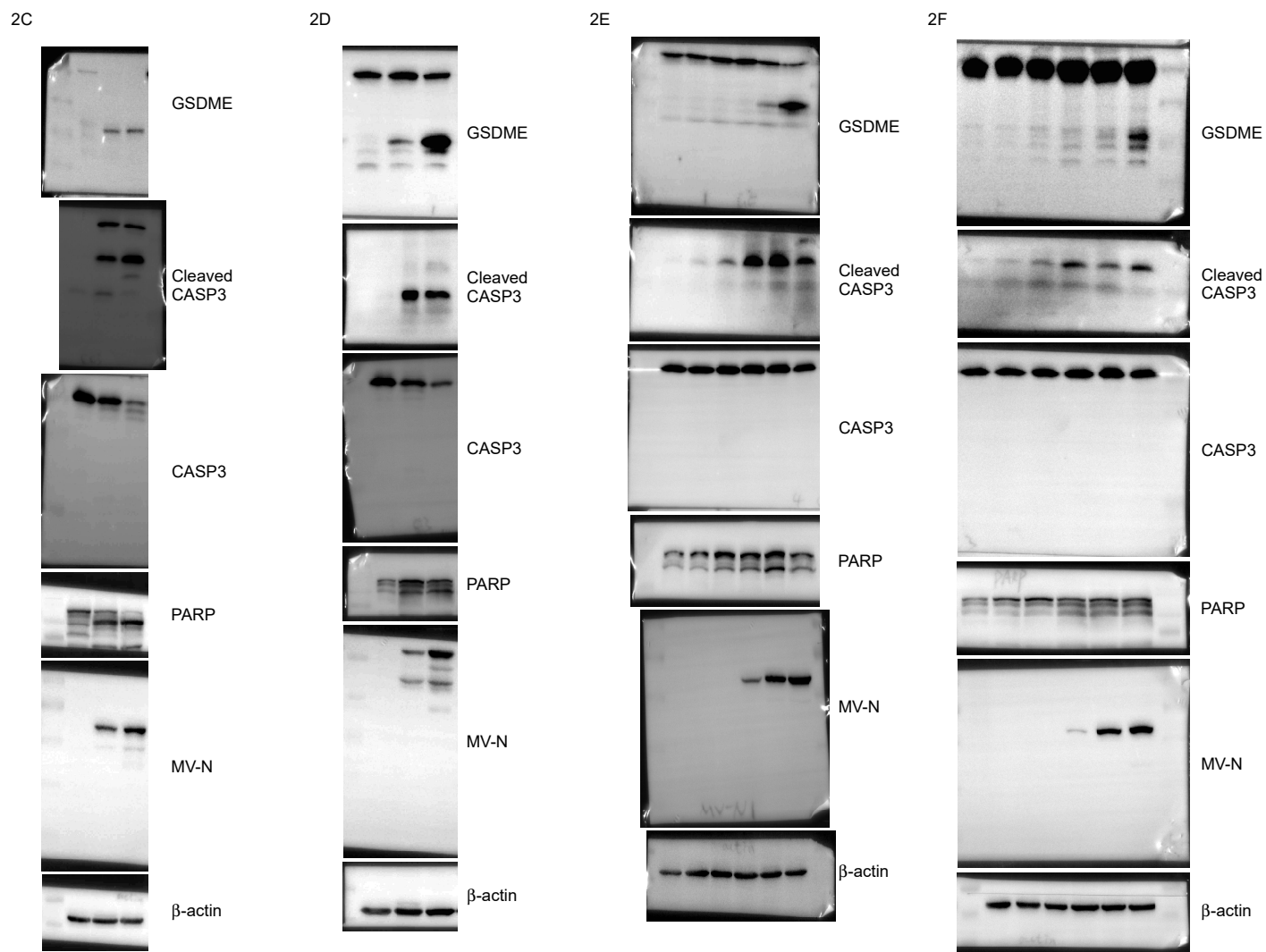

Fig4

4A

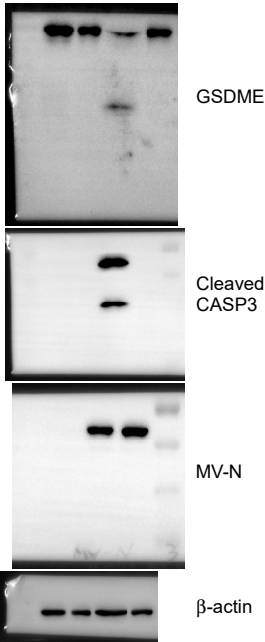

4B

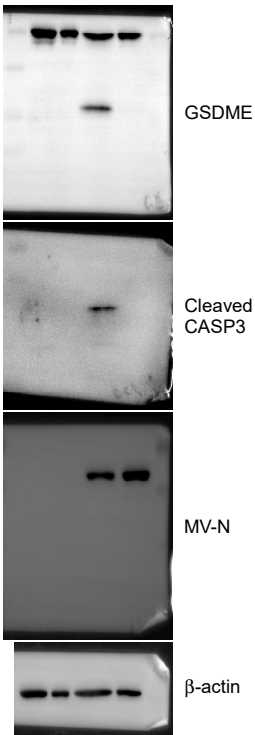

4I

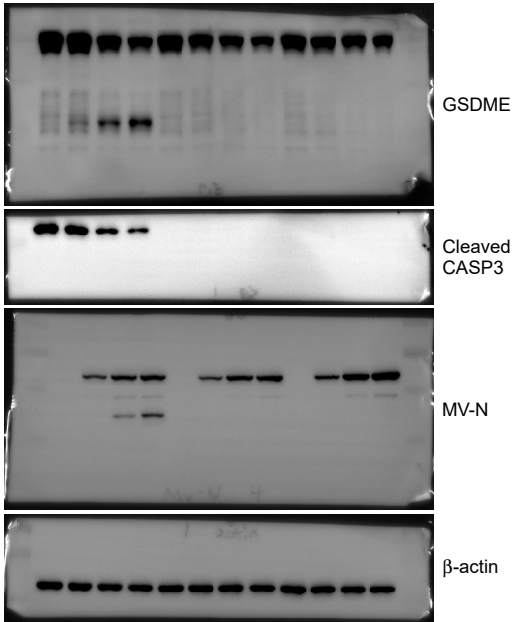

4J

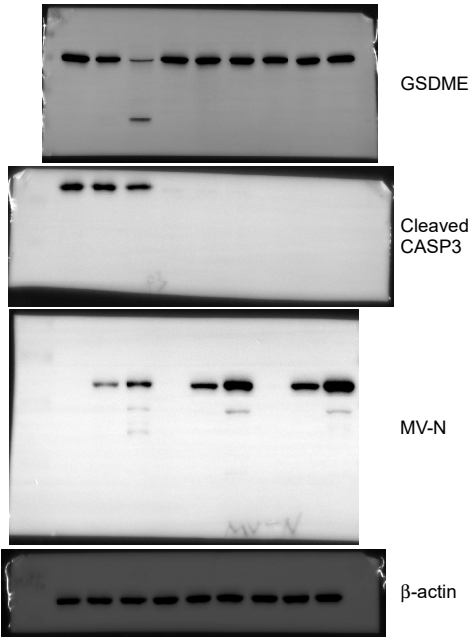

Fig5

5G

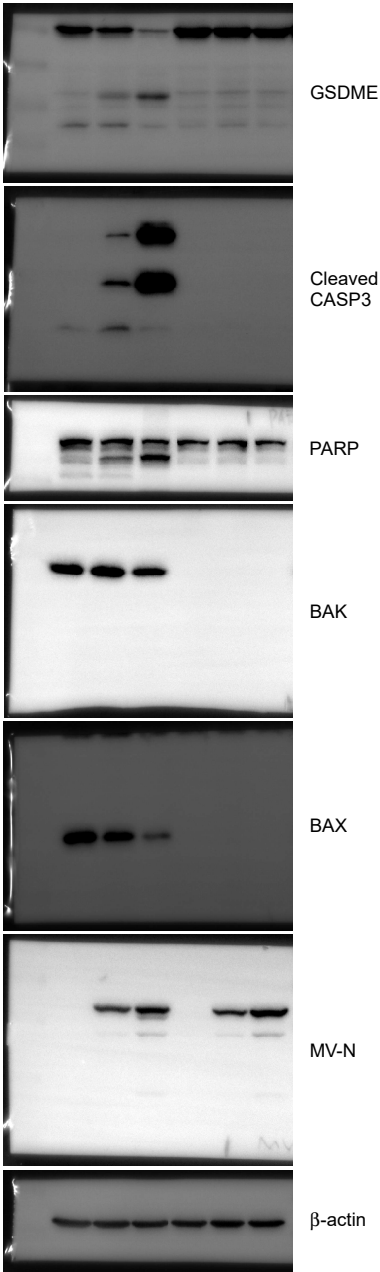

5H

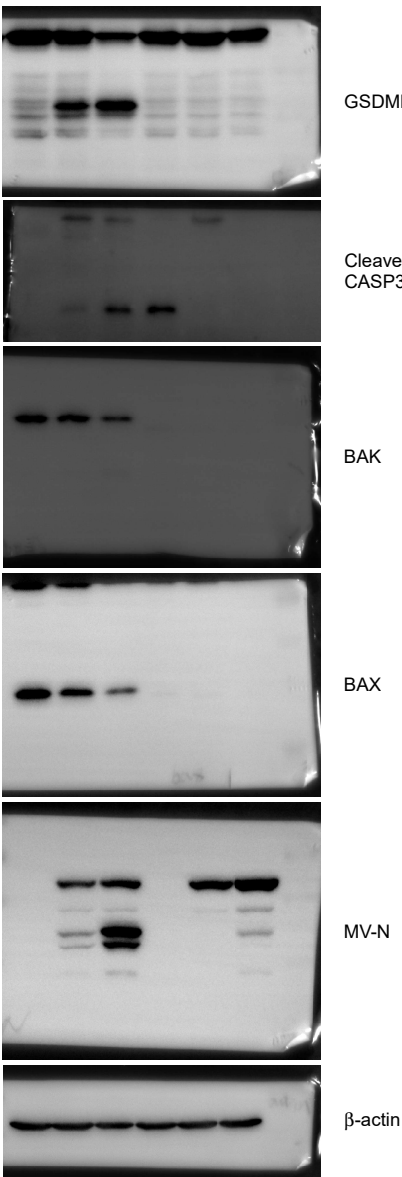

Fig6

6G

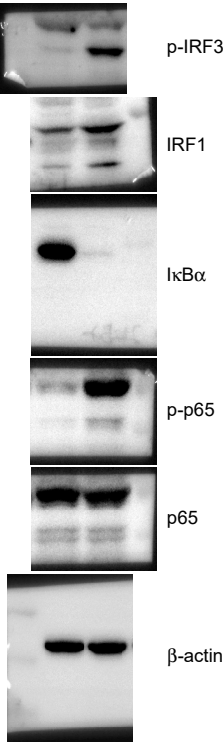

6H

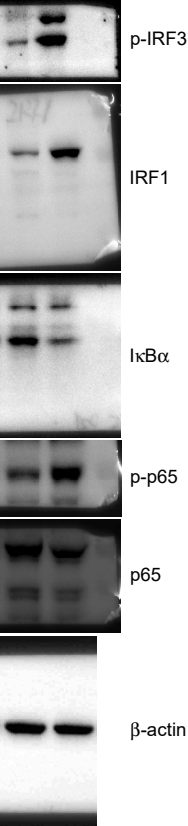

Fig7

7K

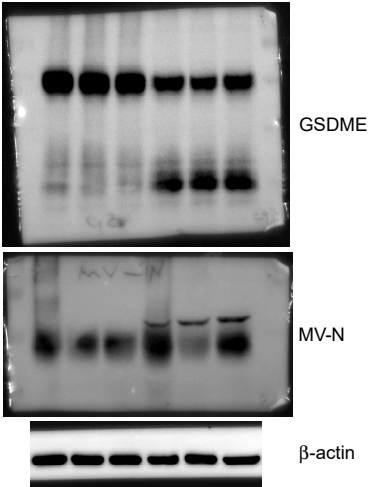

Supplement: Supplementary file 3 — Original Data File [file 41420_2023_1466_MOESM3_ESM.pdf]

Fig S2

S2B

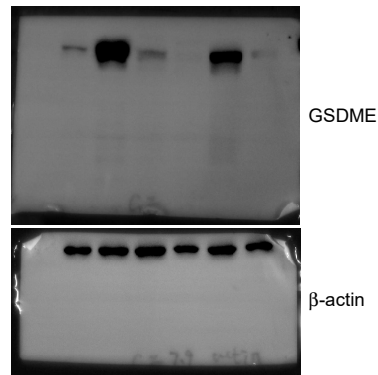

S2C

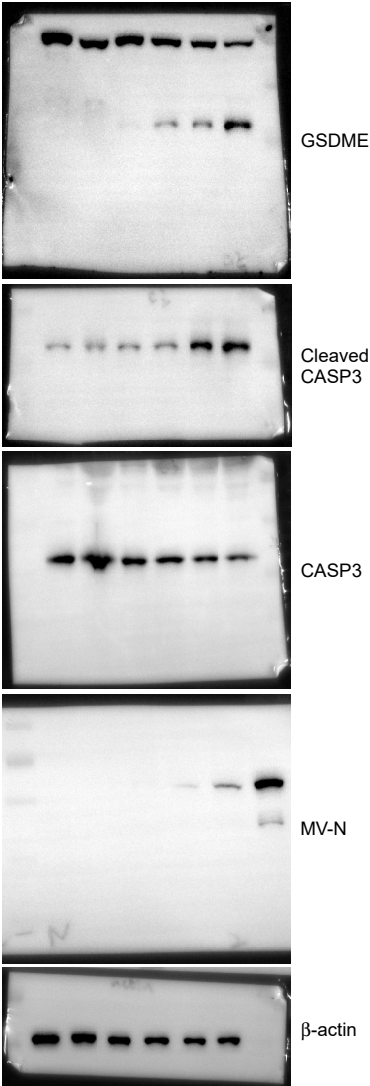

S2D

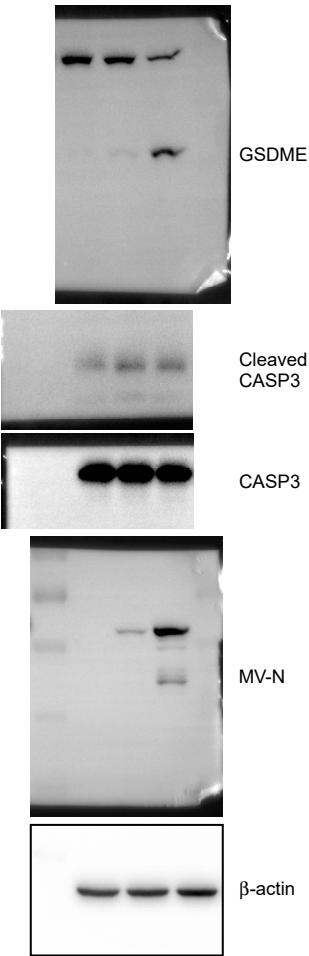

Fig S3

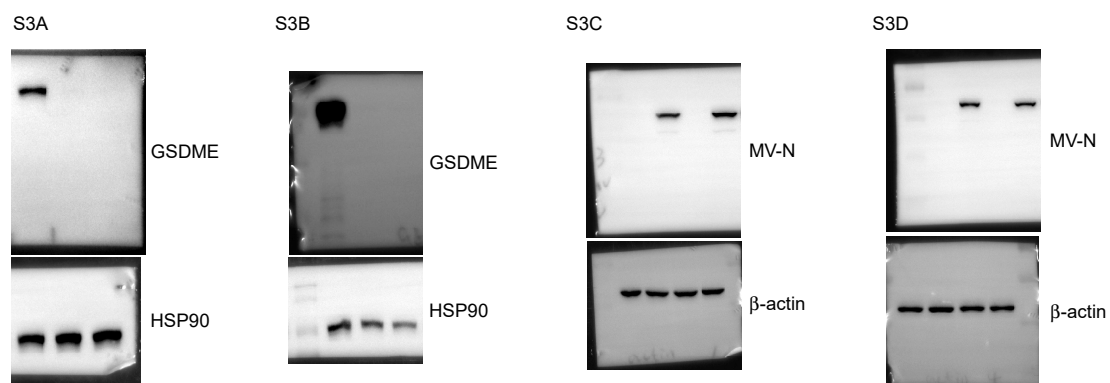

Fig S4

S4B

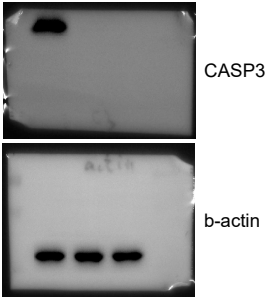

S4C

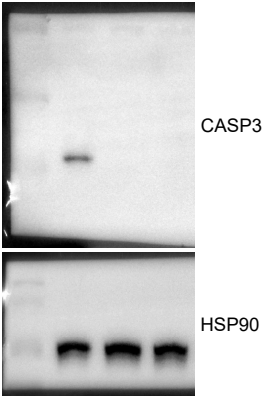

Fig S5

S5E

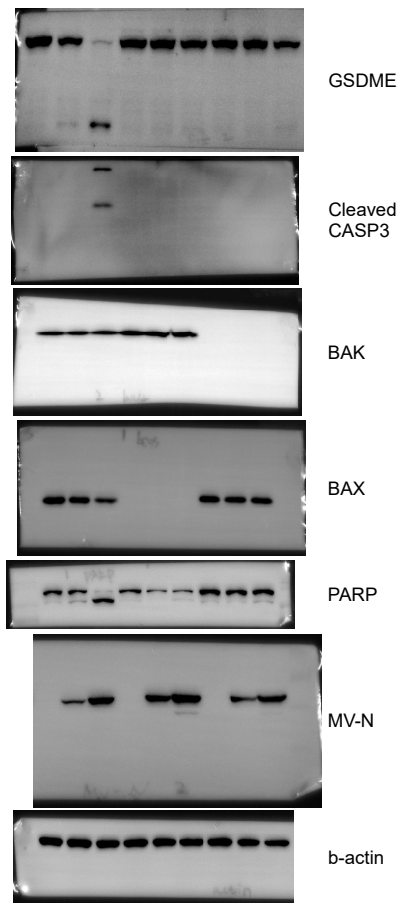

S5F

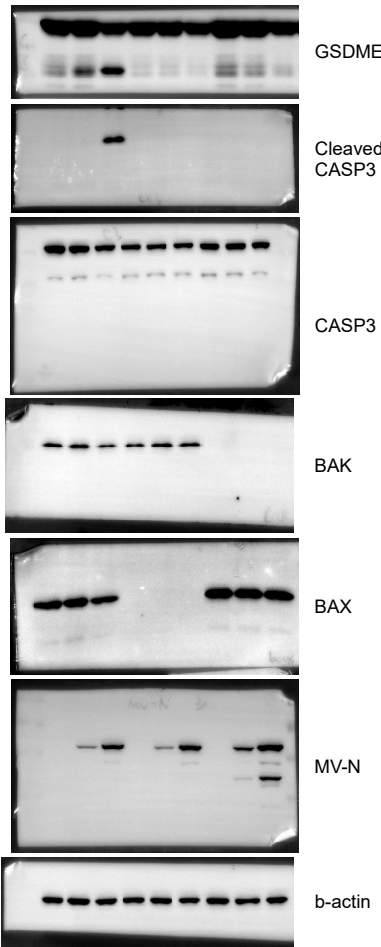

Supplement: Supplementary file 4 — Original Data File [file 41420_2023_1466_MOESM4_ESM.pdf]
